# Comprehensive maternal serum proteomics identifies the cytoskeletal proteins as non-invasive biomarkers in prenatal diagnosis of congenital heart defects

**DOI:** 10.1038/srep19248

**Published:** 2016-01-11

**Authors:** Lizhu Chen, Hui Gu, Jun Li, Ze-Yu Yang, Xiao Sun, Li Zhang, Liping Shan, Lina Wu, Xiaowei Wei, Yili Zhao, Wei Ma, Henan Zhang, Songying Cao, Tianchu Huang, Jianing Miao, Zhengwei Yuan

**Affiliations:** 1 Key Laboratory of Health Ministry for Congenital Malformation, Shengjing Hospital, China Medical University, Shenyang 110004, China; 2 Department of Ultrasound, Shengjing Hospital, China Medical University, Shenyang, China; 3 Department of gynaecology and obstetrics, Shengjing Hospital, China Medical University, Shenyang, China; 4 Department of gynaecology and obstetrics, Shenyang women’s and children’s Hospital, Shenyang, China; 5 Department of genetics, Shenyang Women and Children Health Care Centre, Shenyang, China; 6 Department of Urologic Surgery, Shengjing Hospital, China Medical University, Shenyang, China; 7 Department of Laboratory Medicine, Shengjing Hospital, China Medical University, Shenyang, China; 8 Department of Obstetrics and Gynecology, Eastern Virginia Medical School, Norfolk 23507, USA

## Abstract

Congenital heart defects (CHDs) are the most common group of major birth defects. Presently there are no clinically used biomarkers for prenatally detecting CHDs. Here, we performed a comprehensive maternal serum proteomics assessment, combined with immunoassays, for the discovery of non-invasive biomarkers for prenatal diagnosis of CHDs. A total of 370 women were included in this study. An isobaric tagging for relative and absolute quantification (iTRAQ) proteomic approach was used first to compare protein profiles in pooled serum collected from women who had CHD-possessing or normal fetuses, and 47 proteins displayed significant differential expressions. Targeted verifications were performed on 11 proteins using multiple reaction monitoring mass spectrometry (MRM-MS), and the resultant candidate biomarkers were then further validated using ELISA analysis. Finally, we identified a biomarker panel composed of 4 cytoskeletal proteins capable of differentiating CHD-pregnancies from normal ones [with an area under the receiver operating characteristic curve (AUC) of 0.938, P < 0.0001]. The discovery of cytoskeletal protein changes in maternal serum not only could help us in prenatal diagnosis of CHDs, but also may shed new light on CHD embryogenesis studies.

Congenital heart defects (CHDs), which include malformations of the heart or great vessels, are the most common group of major birth defects, with an incidence of 19–75/1000 live births^1^. Approximately half of the cases are severe, usually requiring one or more surgical procedures in the neonatal period or during childhood^2^, causing a significant burden not only to CHD families but also to society.

Consequently, comprehensive prenatal diagnosis and embryogenesis studies become more and more important. At present, there are no biomarkers used in clinical practice to detect CHDs prenatally. Clinical strategies to diagnose CHDs mainly rely on fetal echocardiography. However, it is limited to a few specialized and experienced centers, thus pregnancies without identified risk factors are not routinely screened. Moreover, the diagnostic accuracy depends on the skills of the screening operators^3^,^4^. Therefore, the detection of biomarkers in maternal blood is accepted as the “holy grail” for prenatal diagnosis. First, it offers the opportunity to eliminate risk-associated invasive procedures, such as amniocentesis and chorionic villus sampling^5^. Second, it could be performed even in the primary hospitals as a screening method, and may help to set indications for referral for fetal echocardiography. Finally, this method probably could provide insight into the pathophysiologic basis for CHDs^6^, which may provide additional information to guide clinical interventions or target therapies before the development of CHDs.

Comparative proteomic analysis is a powerful diagnostic tool to determine the onset, progression, and prognosis of human diseases^7^. It can identify a large number of proteins simultaneously, and protein alterations corresponding to certain pathological conditions at a certain time can be considered in an integrated way, which may be helpful in investigating the mechanism of a biological process^8^. Our research team previously have used two-dimensional gel electrophoresis (2-DE) /mass spectrometry (MS) in discovering neural tube defects (NTDs) -specific biomarkers in amniotic fluid of pregnant rats, and found a number of differentially expressed proteins which were demonstrated to be involved in the embryogenesis of NTDs^8^,^9^. In this study, we used a discovery method (iTRAQ), followed by a verification (MRM-ELISA) pipeline to perform a comparative serum proteomics analysis for identifying maternal circulating biomarkers for prenatal diagnosis of CHDs. To our knowledge, this is the first comprehensive proteomic study aimed at prenatally diagnosing CHDs, and finally we identified a biomarker panel capable of differentiating CHD-pregnancies from normal ones. Surprisingly, these candidates were all involved in the cytoskeleton pathway. CHDs were associated with a specific pattern of changes in nuclear and sarcomeric cytoskeletons, which could have important implications in understanding the mechanisms involved.

## Results

### Identification of differentially expressed serum proteins in the CHD groups using iTRAQ-LC-ESI-MS/MS

**Figure 1:**
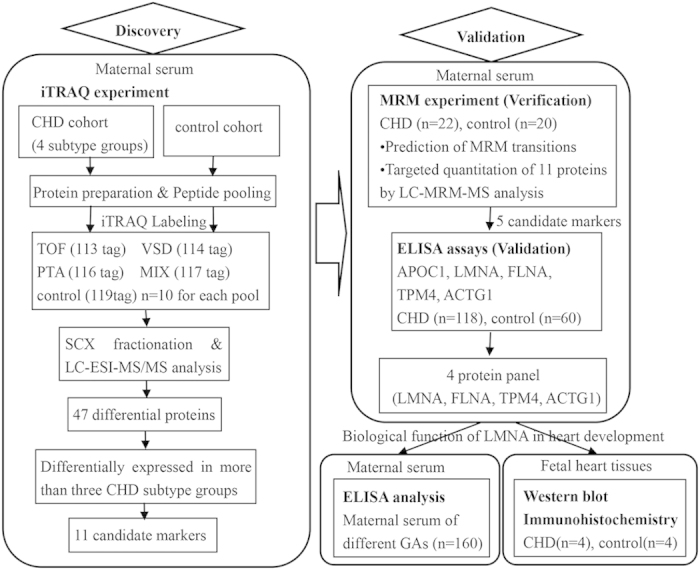
Overall workflow for discovery (iTRAQ) and validation (MRM-ELISA) experiments. TOF, tetralogy of Fallot; VSD, ventricular septal defects; PTA, persistent truncus arteriosus; MIX, a mixture of relatively rare types of CHDs; SCX, Strong Cation Exchange Chromatography; LC-ESI-MS-MS, Liquid chromatography electrospray ionisation tandem mass spectrometry.

Figure 1 shows the workflow leading to the identification of candidate markers. Different types of CHDs may present different protein expression profiles. In the discovery phase, we assigned four CHD subtype pooling groups and one control pooling group (set 1, Table 1). Thus, we could detect not only the differentially expressed proteins between the CHD and control cohorts, but also those specific to various subtypes of CHDs. The first three CHD subtype pools each contained one type of the most common CHDs (TOF, VSD, PTA) (n = 10 for each pool), and the fourth pool was a mixture of relatively rare types of CHDs (n = 10). The control pool contained the same number of samples as the CHD subtype groups. The proteins identified from the four CHD subtype pools were compared with the control pool in a 5-Plex iTRAQ experiment. Comparative protein expression differences were measured by mass spectrometry, and the relative intensities of reporter ions released from each labeled peptide then calculated. Based on the principle that a protein can be identified by at least two unique peptides, a total of 606 proteins from 2549 unique peptides, corresponding to 281606 MS/MS spectra, were identified with 95% confidence (FDR < 3%).

**Figure 2:**
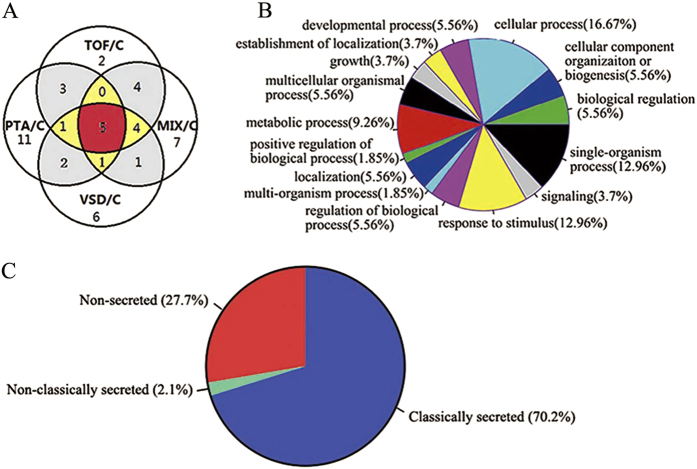
Across the 5-Plex iTRAQ experiment a total of 47 differentially expressed serum proteins were identified. (**A**) Venn diagram: the red region represents five proteins (TPM4, APOC1, SAA2, SAA4, PF4V1) that were differentially expressed in all of the CHD subtype groups, the yellow region represents six proteins (LMNA, MYH9, FLNA, ACTG1, HP, NRX2B) that were differentially expressed in three CHD subtype groups, the grey region represents 10 proteins that were differentially expressed in two CHD subtype groups, and the white region represents 26 proteins that were differentially expressed in one CHD subtype group. (**B**) Classification of the differential expressed proteins in different categories based on their biological functions. TOF, tetralogy of Fallot; VSD, ventricular septal defects; PTA, persistent truncus arteriosus; MIX, a mixture of relatively rare types of CHDs; C, control. (**C**) Distribution of dysregulated proteins predicted as secreted (classically secreted and non-classically secreted) and non-secreted by SignalP and SecretomeP.

Forty-seven differentially expressed proteins were identified based on 1.5-fold overexpression or 1.5-fold underexpression in CHD patients, compared with healthy volunteers (Supplemental Table S1). Twenty-three proteins of the 47 differentially expressed proteins were upregulated in CHD cases, and 24 were downregulated. Some of these proteins were differentially expressed in all CHD subtype groups, while some were differentially expressed in one or more CHD subtype groups (Fig. 2A).

Gene ontology analysis indicated the participation of differentially expressed proteins in a diverse number of biological process, including cellular and single-organism process, response to stimulus, metabolic and developmental process, cellular component organization or biogenesis, biological regulation, localization, etc. (Fig. 2B).

Of 47 dysregulated proteins 33 (70.2%) were predicted to be classically secreted according to the SignalP analysis; and SecretomeP analyses predicted that 1 (2.1%) protein was likely to be non-classically secreted (Fig. 2C).

In addition, we also compared the serum proteomic data among different CHD subtype groups. In the comparison of different subtypes (TOF/VSD, TOF/PTA, TOF/MIX, VSD/PTA, VSD/MIX, and PTA/MIX), there were 18, 18, 19, 20, 18 and 18 dysregulated proteins respectively (Supplemental Fig. S1).

### Targeted quantitation of candidate proteins using MRM-MS

The discovery phase iTRAQ data identified a number of putative biomarkers with implicated roles in the embryogenesis of CHDs. Of these, eleven proteins [Isoform A of Prelamin-A/C (LMNA), Tropomyosin α-4 chain (TPM4), Filamin-A (FLNA), Myosin-9 (MYH9), Apolipoprotein C-I (APOC1), Platelet factor 4 variant (PF4V1), Serum amyloid A-2 protein (SAA2), Serum amyloid A-4 protein (SAA4), Haptoglobin (HP), Actin, cytoplasmic 2 (ACTG1), and Neurexin-2-β (NRX2B)] differentially expressed in more than 3 CHD subtype groups were chosen for further verification using MRM-MS.

More than two high-confidence peptides with a rich product ion spectrum for each target protein were selected for relative quantification analysis. MRM was performed on individual samples in an independent cohort consisting of 20 controls and 22 CHD cases (set 2, Table 1). Only those peptides having more than 2 transitions with the same expression trends could be used for quantitation. Each protein’s intensity was quantitated using the average intensities from its corresponding peptides, and transitions with the best CVs were used for final quantitation of the corresponding peptide. Prior to the statistical analysis, the quantitated protein intensities were log-transformed. The transitions for each peptide are listed in Supplemental Table S2.

**Figure 3:**
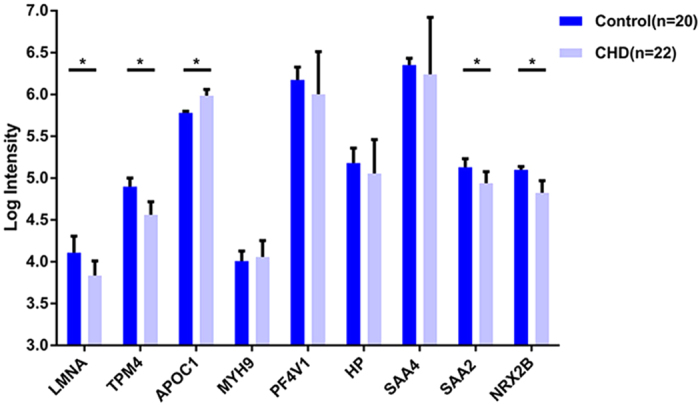
Comparative analysis of expression levels for selected protein candidates for CHD and healthy controls using MRM-MS. *p < 0.05.

Using the established MRM assay, we were able to determine the relative quantity of 9 of the 11 candidates in 42 samples. Quantification of the other two proteins (FLNA, ACTG1) failed because of weak signals. MRM-MS data was exported into skyline V1.4.0.4421, and the peak area of the transition was calculated by normalizing to the area of the internal standard for each sample. Each sample was analyzed in triplicate using MRM, and 9 proteins were monitored showing a CV of 10.4 ± 8.2% (Supplemental Fig. S2). More than 90% of the CVs were below 0.2, and the CVs were relatively high in low concentration points. We noted a significant reduction of LMNA and TPM4 (P = 0.001 and P < 0.001) in the CHD cohorts, with both proteins previously found decreased in the iTRAQ analysis. In addition, APOC1 was significantly upregulated in CHD patients (P = 0.002), which was also consistent with the iTRAQ findings. There were no significant differences in MYH9, PF4V1, HP and SAA4 expression between groups. In contrast to iTRAQ experiments, SAA2 and NRX2B were found downregulated in the CHD cohorts (P = 0.002, and P = 0.010, respectively) (Fig. 3).

### Comparison of measurements obtained by MRM and ELISA analyses

**Figure 4:**
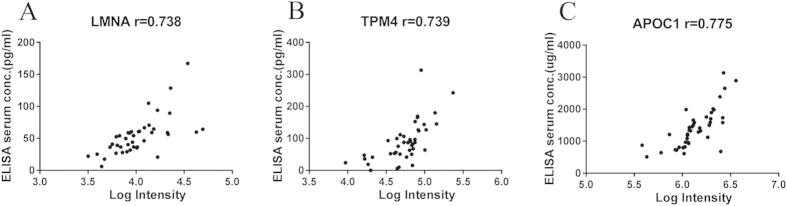
Comparison of LMNA (**A**), TPM4 (**B**), and APOC1 (**C**) levels between MRM and ELISA analyses.

We measured serum levels of LMNA, TPM4 and APOC1 in the same cohort with the MRM experiment (set 2, Table 1). A Corrgram was generated to visualise the correlation between these two methods. Finally, the MRM measurements correlated very well with the ELISA data (p < 0.001) (Fig. 4). This demonstrates that our MRM-based assay provided reliable measurements of relative protein levels across the 42 serum samples.

### Validation of novel protein biomarker candidates using ELISA analysis

**Figure 5:**
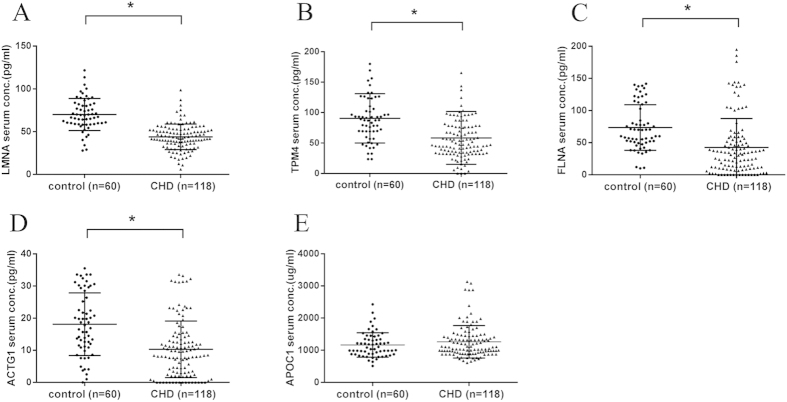
Comparative analysis of expression levels for LMNA **(A)**, TPM4 **(B)**, FLNA **(C)**, ACTG1 **(D)**, and APOC1 **(E)** in maternal serum with CHD and control pregnancies (ELISA assays).

**Figure 6:**
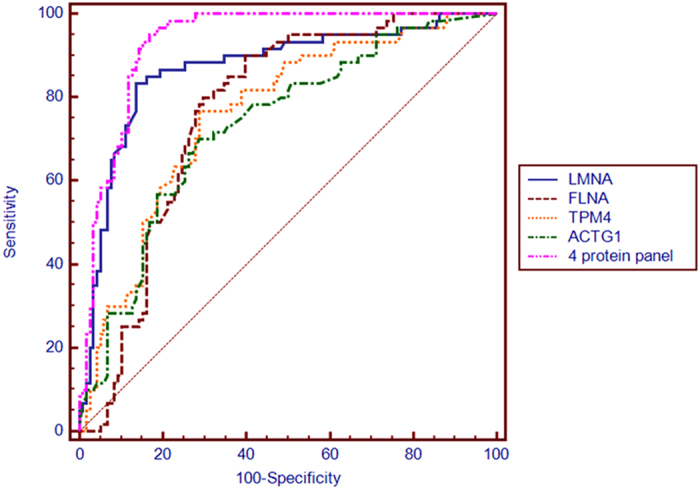
ROC analysis of LMNA, FLNA, TPM4, ACTG1 and combinations to discriminate CHDs from controls (ELISA assays).

The candidate biomarkers verified by the MRM analysis (LMNA, TPM4 and APOC1) were further validated using ELISA assays in a large and independent cohort (set 3, Table 1). The other two proteins (FLNA and ACTG1) failed in the MRM quantitation were also included. The median serum LMNA, TPM4, FLNA, and ACTG1 concentrations were significantly lower in CHD maternal serum, compared with healthy controls (P < 0.001, respectively), while APOC1 showed no significant difference (P = 0.330) (Fig. 5). ROC analysis demonstrated that LMNA, FLNA, TPM4, and ACTG1 could discriminate CHDs from control patients with good statistical power (P < 0.001), and the AUC values were 0.867 (95% CI, 0.807 to 0.927), 0.766 (95% CI, 0.697 to 0.826), 0.760 (95% CI, 0.690 to 0.821), and 0.733 (95% CI, 0.662 to 0.796) respectively. The combination of all four proteins resulted in a robustly increased AUC of 0.938 (95% CI, 0.905 to 0.970), and the sensitivity and specificity were 95.0% (95% CI, 86.1% to 99.0%) and 83.9% (95% CI, 76.0% to 90.0%), respectively (Fig. 6).

### LMNA expression in different stages of normal pregnancy

As a single biomarker, LMNA possessed the best discriminatory power and was thus selected for more detailed analysis. To determine the relationship between serum LMNA concentration and pregnancy, we measured serum LMNA levels at different gestational ages (GAs) in 160 women (set 4, Table 1) with normal fetuses. In the serum of nonpregnant women, LMNA level was very low, with a median of 29.0 pg/ml. During pregnancy, LMNA content began to rise from 42.9 pg/ml (at 7–10 weeks) to 68.1 pg/ml (at 22–26 weeks), before falling gradually to 28.9 pg/ml at GAs of 38–40 weeks (Supplemental Fig. S3).

### Measurement of LMNA in fetal heart tissues

**Figure 7:**
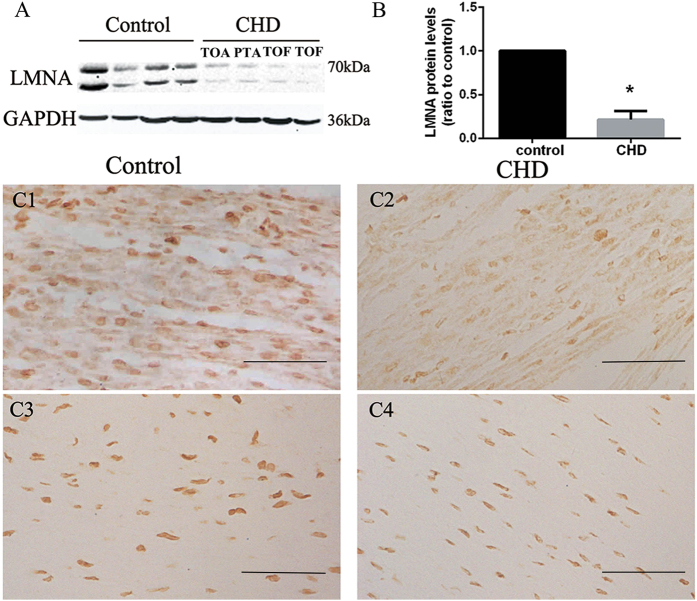
Western blot and IHC analysis confirm reductions of LMNA in heart tisssues of fetuses with CHDs. (**A**,**B**) Western blot analysis showing decreased levels of LMNA in CHD patients. (C1-C4) Distribution of LMNA immunostaining in fetal heart. It was prominent in the myocardium (cytoplasm and nuclear membrane) and great vessles (nuclear membrane) in the normal heart tissues. (C1) Myocardium of normal fetus. (C2) Myocardium of TOF fetus. (C3) Aortic wall of normal fetus. (C4) Aortic wall of TOF fetus. Scale bars: 20 μm.

Consistent with the serum results, the expression of LMNA in fetal heart tissues was significantly lower in the CHD group than the control group, as measured by Western blotting and IHC (Fig. 7). IHC showed LMNA expression in fetal heart myocardium, as well as in great vessels and endocardial tissues. However, the expression of LMNA was different among these tissues. In the myocardial tissues, LMNA immunostaining was prominent in both the cytoplasm and the nuclear membrane. In contrast, LMNA immunoreactivity localized only to the nuclear membrane in great vessels and endocardial tissues.

## Discussion

Comparative proteomic analysis recently has been used in prenatal diagnosis of fetal abnormalities, and found a number of differentially expressed proteins for non-invasive diagnosis of preeclampsia^10^,^11^ and chromosome disorders^12^,^13^,^14^,^15^. To our knowledge, this is the first comprehensive proteomic study of maternal serum with CHD fetuses. Using this technique, we identified a panel consisting of 4 proteins (LMNA, FLNA, TPM4, and ACTG1) with fair robustness in regard to specificity and sensitivity in differentiating CHDs from controls. Dysregulation of these proteins not only enabled the use of maternal serum for discovery of candidate biomarker proteins in prenatal diagnosis of CHDs but also reflect the molecular origins of CHDs.

Although emerging proteomics profiling technologies have been used in discovering new biomarkers, successful applications to human disease are still lacking. This is due, in large part, to the lack of a coherent, demonstrably successful pipeline from the discovery to the verification stage^16^. In this study, the success of the proteomics pipeline largely depends on the following aspects. First, all of the serum specimens were collected and processed consistently according to the strict method described above. Thus, the quality of samples and consequently the resulting data are reliable^17^. In the discovery period, we performed shotgun quantitative proteomics first using iTRAQ labeling with pooled serum collected from women who had CHD or event-free pregnancies. iTRAQ has a clear-cut advantage over other existing proteomic techniques in that it allows quantitation and multiplexing in a single experiment, and could be used in a wide range of biological samples, such as cells and tissue, and body fluids such as serum and plasma^6^. Our study revealed 47 serum proteins that were differentially expressed in the CHD cohorts. We chose proteins differentially expressed in more than 3 CHD subtype groups for further verification; thus, the candidate biomarkers may have the potential to detect various subtypes of CHDs. During the verification stage, MRM was performed first to identify false positives from the discovery phase and allow prioritisation of proteins to be taken into large-scale clinical validation studies^18^,^19^,^20^. Since CHDs are a group of polygenetic diseases, as with any multigenic process, there is an important role for high-throughput methods to characterize the genes and proteins involved in disease processes^21^. MRM-MS is non-antibody-based, and enables us to reliably quantify tens to hundreds of candidate biomarker proteins at the same time. Finally, the proteins confirmed by MRM were further evaluated in a large and independent cohort using ELISA analysis, which is the current gold standard for biomarker validation and clinical diagnostics^22^. Consequently, the proteins identified through the above profiles may have a potential value as novel predictive markers for CHDs.

In the 47 dysregulated proteins identified in this study, 72.3% were secretory proteins, and 17.0% were cytoskeletal proteins. We chose proteins differentially expressed in more than 3 CHD subtype groups for further verification. Coincidentally, the candidate biomarkers obtained after validation all belong to the cytoskeleton family. These cytoskeletal proteins are probably pregnancy-specific, as our study found that serum concentration of LMNA was very low in nonpregnant women and began to rise during pregnancy. Similar to our findings, Scholl *et al*. studied maternal serum proteome changes between the first and third trimester of pregnancy, and observed that the serum concentration of gelsolin (belong to the cytoskeleton family) changed during pregnancy^23^. Besides, a cluster of cytoskelatal proteins were found to be less abundant in the serum of women with Down syndrome-pregnancies and preeclampsia^13^,^14^,^24^, and 40% of Down syndrome fetuses have CHDs^25^. All above indicated that dysregulation of these cytoskeletal proteins might reflect disturbance of embryonic development, thus, deep exploration of these protein changes in maternal serum may facilitate prenatal diagnosis of pregnancy-related disorders.

The exact mechanism for the downregulation of cytoskeletal proteins is still unclear. The levels of LMNA in CHD fetal heart tissues were significantly lower than those in normal controls, so it is plausible that the decreased LMNA in the CHD fetus’ circulation is a potential source of that in maternal serum. On the other hand, Cole *et al*. proposed CHDs might be linked to maternal autoimmunity against certain cytoskeletal proteins. The maternally transferred antibodies could affect fetal cytoskeletal protein synthesis and cardiomyocyte proliferation^26^. Similarly, we found some proteins (CD5L, CD14, and properdin) involved in immuno-rejection^27^,^28^,^29^ were upregulated in the iTRAQ experiment. Thus, further studies will be needed to dectect whether the downregulation of cytoskeletons was attributed to the maternal autoimmunity.

Although we have not fully understood why these cytoskeletal proteins were downregulated in maternal serum of CHD cohort, it has been demonstrated that these proteins are essential for the structure and function of the cardiac myocyte and have been shown to be altered in heart failure and/or cardiomyopathy^30^,^31^,^32^,^33^. Our study found that CHDs were associated with specific changes in the expression of nuclear and sarcomeric cytoskeletons. FLNA, TPM4, and ACTG1 belong to the sarcomeric proteins. FLNA is an actin-binding protein that crosslinks actin into orthogonal networks and interacts with a variety of other proteins, including membrane proteins, integrins, transmembrane receptor complexes, and second messengers, thus forming an important intracellular signaling scaffold^34^. Complete loss of FLNA in mice results in embryonic lethality with severe cardiac structural defects involving ventricles, atria, and outflow tracts, as well as widespread aberrant vascular patterning^35^. TPM4 is an actin-filament binding protein that plays important roles in modulating muscle contraction^36^, and is essential for heartbeat in zebrafish embryos^37^. ACTG1 is a cytoplasmic actin playing fundamental roles in most cellular processes, including muscle contraction, cell motility, cell division, cell signaling, establishment of cell polarity and maintenance of cell shape. ACTG1^−/−^ mice exhibited stunted growth during embryonic and postnatal development as well as delayed cardiac outflow tract formation that resolved by birth^38^.

LMNA is the main constituent of the nuclear cytoskeleton^39^. It acts as a scaffold for protein complexes that regulate nuclear structure and functions^40^, and may participate in signal transduction by mediating movement between the cytoplasm and the nucleus^41^. We found decreased LMNA levels in heart tissues of CHD fetuses, implying a potential role of LMNA in fetal heart development. Upon differentiation to neuronal lineages and cardiomyocytes, human embryonic stem cells begin to express LMNA^42^. Once expressed, LMNA could “lock” the nucleus of differentiated cells in particular gene expression patterns^43^, raising the possibility that CHDs occur because cells cannot maintain their differentiated state due to a disorganized nuclear lamina caused by LMNA deficiency. The expression pattern of LMNA at different GAs indicate that LMNA is a pregnancy associated marker and possibly derived from fetus. We established a reference range for maternal serum LMNA at different GAs for the first time, which may lay a foundation for early prenatal diagnosis.

Therefore, the functions of LMNA, FLNA, TPM4 and ACTG1 support these proteins as general markers for CHDs. We suspect that the interactions between the nucleoskeleton and sarcomaric skeleton may play an critical role in the CHD embryogenesis. Ho *et al*. presented a novel mechanism that in many laminopathies, lamin A/C regulate gene expression through modulation of nuclear and cytoskeletal actin polymerization^44^. Nikolova-Krstevski *et al*. found that in dilated cardiomyopathy, LMNA interacted with Nesprins, which serves as the linker-of-nucleoskeleton-and-cytoskeleton complex connecting the nuclear envelope to the actin cytoskeleton^45^. Although the precise pathophysiological role of these cytoskeletal proteins in CHDs remains unclear, we hypothesize that there is a nucleoskeleton-and-sarcomaric skeleton pathway in the heart development.

Currently, fetal echocardiography is the major tool for CHD prenatal diagnosis, and the accurate diagnosis highly depends on the skills of the operators. Furthermore, many primary hospitals in China can not develop the routine fetal ultrasound scan and echocardiography. Therefore, there is an increasing demand for the development of a new method for CHDs screening. Proteomic profiles enabled us to detect proteins differentially expressed between CHD and control cohorts, and to reliably quantify tens to hundreds of proteins at the same time. This is especially suitable for screening polygenetic diseases such as CHDs, and the biomarker panel composed of several proteins obtained through the above procedures have high sensitivity and specificity. It has such advantages as rapid measurement, accuracy, simplicity of use, and the detection of these proteins from maternal serum is non-invasive, requires small amounts of sample, thus it could be provided to all pregnant women even in the primary hospitals. Besides, this method probably could detect serum changes predicted CHDs in an earlier period, which may provide additional information to guide clinical interventions before the development of CHDs.

## Methods

### Participants

A total of 370 women were included in this study. The general characteristics of the study population are provided in Table 1. Samples used in the iTRAQ experiment (n = 50) and ELISA analysis (n = 178) were collected at Shengjing Hospital of China Medical University from 2011 to 2014 (set 1 & set 3), and samples used for MRM-MS analysis (n = 42) were collected from Shenyang Women and Children Health Care Centre from 2011 to 2013 (set 2). The maternal serum samples were collected at 22 to 26 weeks, and the diagnoses of CHDs were confirmed by fetal echocardiography and/or autopsy and/or postnatal surgery. All women remained healthy throughout their pregnancies. In addition, we randomly selected 80 women (four groups with n = 20) carrying normal fetuses with GAs of 8–10w, 16–19w, 32–34w, and 38–40w. Twenty nonpregnant and healthy women were also included (set 4). The serum samples in set 4 were collected at Shengjing Hospital of China Medical University, and these samples were used to detect LMNA concentrations at different GAs of normal pregnancies.

Fetal heart samples from four abortive CHD fetuses and four abortive fetuses without heart defects were also collected at Shengjing Hospital of China Medical University. In particular, samples with GAs of 22 to 26 weeks were paired based on age and gender. The medical information of eight fetuses is summarized in Table 1 (set 5).

Informed consent was collected from the volunteers, conforming to the Declaration of Helsinki, was approved by the Ethics Committee of Shengjing Hospital and Shenyang Women and Children Health Care Centre. All experiments were carried out in accordance with the approved guidelines.

### Sample preparation

Serum was harvested immediately after blood collection from each woman. In brief, 5-mL venous blood samples were collected by separating gel vacuum tubes and centrifuged at 3000 rpm at 4 °C for 15 minutes. Serum layers were aliquotted and stored at −80 °C. Samples were processed within 4 hours of collection. Stored serum samples were defrosted on ice and maintained below 4 °C during experiments, whenever possible.

All heart tissue samples were stored at −80 °C until analysis. Tissue samples suitable for Western blot analyses were collected from left ventricles. Additional tissue samples were fixed in 4% paraformaldehyde and embedded in wax, and 3-μm-thick sections were processed for immunohistochemistry.

### iTRAQ discovery experiments

Equal volumes of serum samples from 10 pregnant women in each group were pooled for iTRAQ analysis. Pooled serum samples were depleted of high-abundance proteins using ProteoMiner Protein Enrichment Kits (Bio-Rad Laboratories, Inc., USA) according to the manufacturer’s instructions. Following protein preparation and digestion, peptides were labeled with iTRAQ reagents, fractionated, and analyzed by Liquid chromatography electrospray ionisation tandem mass spectrometry (LC-ESI-MS/MS). A detailed description of the methods is provided in the online Supplemental material.

### Database search

Proteins identification were performed using Mascot search engine (version 2.3.02; Matrix Science, London, UK) against the International Protein Index (IPI) databases (version 3.87, HUMAN) containing 91464 sequences. For protein identification, a mass tolerance of 0.05 Da was permitted for intact peptide masses and 0.1 Da for fragmented ions, with allowance for one missed cleavages in the trypsin digests. Variable modifications included Gln- > pyro-Glu (N-term Q), Oxidation (M), iTRAQ8plex (Y), and fixed modifications included Carbamidomethyl (C), iTRAQ 8 plex (N-term), iTRAQ 8 plex (K). The charge states of peptides were set to +2 and +3. To reduce the probability of false peptide identification, only peptides with significance scores (≥20) at the 99% confidence interval by a Mascot probability analysis greater than “identity” were counted as identified. For protein quantitation, it was required that a protein contains at least two unique peptide. The quantitative protein ratios were weighted and normalized by the median ratio in Mascot. We only used ratios with p-values <0.05, and only fold changes of >1.5 were considered as significant.

### LC-MRM-MS analysis

MRM-MS was performed in individual samples on an independent cohort (set 2, Table 1) consisting of 22 CHD and 20 control subjects. High-confidence peptides of the target proteins exhibiting rich product ion spectra were selected for MRMs. The analyses for all the experiments were performed on a QTRAP 5500 mass spectrometer instrument (AB SCIEX, Foster City, CA, USA) equipped with a Waters nano Acquity Ultra Performance LC system. We detected and verified transitions by MRM + Enhanced Product Ion (EPI) mode. ProteinPilot software (Applied Biosystems) was used to search protein database for MS/MS data generated from the MRM-MS assays. Twenty-four femtomoles of β-Galactosidase peptides were added to each of the 42 samples as internal standards^46^,^47^. Each of these samples were prepared in triplicate and processed in a randomized order. These replicate sample preparations were used to determine the CV of the MRM assay for each peptide. For detailed experimental procedures, see the online Supplemental material.

### ELISA assays

The biomarker candidates were validated by ELISA assays in a large cohort (set 2 and set 3, Table 1). In addition, the serum concentrations of LMNA at different GAs were measured (sets 4, Table 1). ELISA measurements for APOC1 (RayBiotech, ELH-ApoC1, USA), LMNA (CUSABIO, EL013003HU, China), TPM4 (CUSABIO, EL024108HU, China), FLNA (CUSABIO, EL008724HU, China), and ACTG1 (CUSABIO, EL001222HU, China) were performed according to the manufacturers’ instructions. All assays were performed in triplicate.

### Western Blot Analyses and Immunohistochemistry

Western blot analyses and immunohistochemistry (IHC) were performed with antibodies against human LMNA using fetal heart tissues. Details of these methods are provided in the online Supplemental material.

### Statistical Analysis

Results were expressed as mean ± SD or median with inter-quartile range. Student’s two-sided t-test was used for normally distributed values. For variables without normal distribution, Mann-Whitney U test was performed. Spearman correlation coefficients were used for correlation analysis. Receiver operating characteristic curves (ROCs) were analyzed to assess specificity and sensitivity of single candidate biomarkers and their combinations using binary logistic regression analysis.

Statistical analyses were performed using SPSS 20.0, MedCalc 11.4.2.0, and GraphPad Prism 6.0. P < 0.05 was considered statistically significant.

## Additional Information

**How to cite this article**: Chen, L. *et al*. Comprehensive maternal serum proteomics identifies the cytoskeletal proteins as non-invasive biomarkers in prenatal diagnosis of congenital heart defects. *Sci. Rep*. **6**, 19248; doi: 10.1038/srep19248 (2016).

## Supplementary Material

Supplementary Information

## Figures and Tables

**Table 1:** Clinical characteristics of the study population.

Discovery set (iTRAQ 5-Plex) (set 1) (n = 50)									
	Control (n = 10)	TOF^a^ (n = 10)		VSD^b^ (n = 10)		PTA^c^ (n = 10)		MIX^d^ (n = 10)	
MA^e^ (y)	28.0 ± 2.7	29.0 ± 2.9		29.2 ± 1.6		27.8 ± 5.3		29.9 ± 2.9	
GA^f^ (w)	24.0 ± 0.7	23.8 ± 0.8		24.4 ± 0.9		24.8 ± 2.2		24.2 ± 1.0	
**MRM-Validation set (set 2) (n = 42)**									
	Control (n = 20)	CHD (n = 22)							
		TOF (n = 4)	VSD (n = 4)	PTA (n = 4)	TOA^g^ (n = 4)	HLHS^h^ (n = 3)		SV^i^ (n = 3)	
MA	27.1 ± 5.0	30.6 ± 2.6	28.4 ± 1.7	29.0 ± 2.9	28.0 ± 1.1	28.7 ± 5.1		27.7 ± 4.7	
GA	24.2 ± 1.4	24.2 ± 1.6	23.4 ± 1.1	24.0 ± 1.4	24.4 ± 1.7	24.7 ± 2.3		23.3 ± 1.5	
**ELISA-Validation set (set 3) (n = 178)**									
	Control (n = 60)^▲^	CHD (n = 118)							
		TOF (n = 41)	VSD (n = 21)	TOA (n = 13)	PTA (n = 12)	HLHS (n = 10)	SV (n = 6)	PS^j^ (n = 6)	OT^k^ (n = 9)
MA	28.4 ± 4.2	29.8 ± 4.8	27.3 ± 4.0	27.1 ± 3.7	28.0 ± 3.9	26.4 ± 5.4	29.0 ± 3.8	28.8 ± 3.8	28.3 ± 3.9
GA	24.3 ± 1.4	24.5 ± 1.5	23.6 ± 1.4	24.6 ± 1.1	24.3 ± 1.5	24.5 ± 1.3	23.7 ± 1.0	24.7 ± 1.2	23.9 ± 1.3
**Different GA set (normal pregnancy) (set 4) (n = 160)**									
	8–10w (n = 20)	16–19w (n = 20)	22–26w (n = 60)^▲^	32–34w (n = 20)		38–40w (n = 20)		NP^l^ (n = 20)	
MA	29.4 ± 4.9	28.9 ± 3.2	28.4 ± 4.2	29.1 ± 4.4		29.3 ± 3.9		29.5 ± 3.0	
GA	9.1 ± 0.8	17.6 ± 1.1	24.3 ± 1.4	32.8 ± 0.8		38.6 ± 0.7		0	
**Heart tissue specimens (set 5) (n = 8)**									
	CHD cohort				Control cohort				
Defects	TOA	PTA	TOF	TOF	Control 1	Control 2	Control 3	Control 4	
MA	28	30	26	25	24	26	27	30	
GA	23	25	25	26	22	24	25	26	
Gender	female	male	male	female	female	male	male	female	

^a^Tetralogy of Fallot.

^b^Ventricular septal defects.

^c^Persistent truncus arteriosus.

^d^Mixture of relatively rare types of CHDs (e.g., single ventricle, hypoplastic left heart syndrome, endocardial cushion defect, aortic stenosis, cardiac rhabdomyoma).

^e^Maternal age.

^f^Gestational age.

^g^Transposition of the great arteries.

^h^Hypoplastic left heart syndrome.

^i^Single ventricle.

^j^Pulmonary artery valve stenosis.

^k^Other defects (e.g., endocardial cushion defect, aortic stenosis, cardiac rhabdomyoma, total anomalous pulmonary venous connection, and levocardia).

^l^Nonpregnant. ^„▲„^ represent the same cohort.
