# Supplementary material for: Comprehensive maternal serum proteomics identifies the cytoskeletal proteins as non-invasive biomarkers in prenatal diagnosis of congenital heart defects
Source: Sci Rep. 2016 Jan 11;6:19248. doi: 10.1038/srep19248 (PMC4707500; doi:10.1038/srep19248)
Supplement: Supplementary Information [file srep19248-s1.doc]

# Comprehensive maternal serum proteomics identifies the cytoskeletal proteins as non-invasive biomarkers in prenatal diagnosis of congenital heart defects

Lizhu Chen^1,2^, Hui Gu^1^, Jun Li^3^, Ze-Yu Yang^2^, Xiao Sun^4^, Li Zhang^5^, Liping Shan^6^, Lina Wu^7^, Xiaowei Wei^1^, Yili Zhao^8^, Wei Ma^1^, Henan Zhang^1^, Songying Cao^1^, Tianchu Huang^1^, Jianing Miao^1^＆Zhengwei Yuan^1*^

^1^Key Laboratory of Health Ministry for Congenital Malformation, Shengjing Hospital, China Medical University, Shenyang 110004, China. ^2^Department of Ultrasound, Shengjing Hospital, China Medical University, Shenyang, China. ^3^Department of gynaecology and obstetrics, Shengjing Hospital, China Medical University, Shenyang, China. ^4^Department of gynaecology and obstetrics, Shenyang women's and children's Hospital, Shenyang, China. ^5^Department of genetics, Shenyang Women and Children Health Care Centre, Shenyang, China. ^6^Department of Urologic Surgery, Shengjing Hospital, China Medical University, Shenyang, China. ^7^Department of Laboratory Medicine, Shengjing Hospital, China Medical University, Shenyang, China. ^8^Department of Obstetrics and Gynecology, Eastern Virginia Medical School, Norfolk 23507, America.

Correspondence and requests for materials should be addressed to Z.Y. (yuanzw@hotmail.com)

**Supplementary Information**

**Supplemental Methods**

**iTRAQ labelling, peptide fractionation and LC-ESI-MS/MS analysis**

**Protein preparation**

Pooled serum samples were depleted of high-abundance proteins using ProteoMiner Protein Enrichment Kits (Bio-Rad Laboratories, Inc., USA) according to the manufacturer’s instructions. The proteins were reduced with 10 mM DL-Dithiothreitol (DTT) (final concentration) at 56°C for 1 h and then alkylated by 55 mM iodacetamide (IAM) (final concentration) in the darkroom for 45min. The reduced and alkylated protein mixtures were precipitated by adding 5× volume of chilled acetone at -20 °C overnight. After centrifugation at 4 °C, 30 000g, the pellet was dissolved in 0.5 M tetraethyl-ammonium bromide (TEAB) (Applied Biosystems, Milan, Italy) and sonicated in ice. After centrifuging at 30 000g at 4 °C, an aliquot of the supernatant was taken for determination of protein concentration. The proteins in the supernatant were kept at -80°C for further analysis. The total protein concentration was calculated by Bradford assay (Bio-Rad Protein Assay; Bio-Rad Laboratories, Hercules, CA, USA).

**iTRAQ Labeling and SCX fractionation**

Total protein (100μg) was taken out of each sample solution and then the protein was digested with Trypsin Gold (Promega, Madison, WI, USA) with the ratio of protein: trypsin =20:1 at 37°C for 12 hours. After trypsin digestion, peptides were dried by vacuum centrifugation. Peptides were reconstituted in 0.5M TEAB and processed according to the manufacture’s protocol for 8-plex iTRAQ reagent (AB Sciex, Framingham, MA, USA). Samples were labeled with the iTRAQ tags as follow: control (119 tag), TOF (113 tag), VSD (114 tag), PTA (116 tag), MIX (117 tag). The peptides were labeled with the isobaric tags, incubated at room temperature for 2h. The labeled peptide mixtures were then pooled and dried by vacuum centrifugation.

SCX chromatography was performed with a LC-20AB HPLC Pump system (Shimadzu, Kyoto, Japan). The iTRAQ labeled peptide mixtures were reconstituted with 4 mL buffer A (25 mM NaH_2_PO_4_ in 25% ACN, pH 2.7) and loaded onto a 4.6×250 mm Ultremex SCX column containing 5-μm particles (Phenomenex). The peptides were eluted at a flow rate of 1mL/min with a gradient of buffer A for 10 min, 5-35% buffer B (25mM NaH_2_PO_4_, 1 M KCl in 25% ACN, pH 2.7) for 11 min, 35-80% buffer B for 1 min. Elution was monitored by measuring the absorbance at 214 nm, and fractions were collected every 1 min. The eluted peptides were pooled into 20 fractions, desalted with a Strata X C18 column (Phenomenex) and vacuum-dried.

**LC-ESI-MS/MS analysis based on Triple TOF 5600**

A splitless nanoACQuity (Waters, USA) system coupled with Triple TOF was used for analytical separation. Microfluidic traps and nanofluidic columns packed with Symmetry C18 (5 μm, 180 μm × 20 mm) were utilized for online trapping, desalting, and nanofluidic columns packed with BEH130 C18 (1.7 μm, 100 μm ×100 mm) were employed in analytical separation. Solvents purchased from Thermo Fisher Scientific (USA) were composed of water/acetonitrile/formic acid (A: 98/2/0.1%; B: 2/98/0.1%). A portion of a 2.25 μg (9 μL) sample was loaded, and trapping and desalting were carried out at 2 μL/min for 15 min with mobile phase A. At a flow rate of 300 nL/min, analytical separation was established by maintaining 5% B for 1 min, then the 40min linear gradient was run starting from 5 to 35% B, followed by 5 min linear gradient to 80%, and maintained for 5 min. Initial chromatographic conditions were restored in 2 min.

Data acquisition was performed with a Triple TOF 5600 System (AB SCIEX, Concord, ON) fitted with a Nanospray III source (AB SCIEX, Concord, ON) and a pulled quartz tip as the emitter (New Objectives, Woburn, MA). Data was acquired using an ion spray voltage of 2.5 kV, curtain gas of 30 psi, nebulizer gas of 15 psi, and an interface heater temperature of 150°C. The MS was operated with a resolution power (RP) of greater than or equal to 30, 000 full width at half maximum (FWHM) for TOF MS scans. For information dependent acquisition (IDA), survey scans were acquired in 250 ms and as many as 30 product ion scans were collected if exceeding a threshold of 120 counts per second (counts/s) and with a 2+ to 5+ charge-state. Total cycle time was fixed to 3.3s. Q2 transmission window was 100Da for 100%. Four time bins were summed for each scan at a pulser frequency value of 11 kHz through monitoring of the 40 GHz multichannel time-to-digital converter (TDC) detector with four-anode channel detect ion. A sweeping collision energy setting of 35±5 eV coupled with iTRAQ adjust rolling collision energy was applied to all precursor ions for collision-induced dissociation. Dynamic exclusion was set for 1/2 of peak width (18s), and then the precursor was refreshed off the exclusion list.

**LC-MRM-MS Method development**

Multiple reaction monitoring (MRM) was performed to verify the identified rubber particle proteins. Eleven rubber particle proteins were selected for MRM analysis in this research. The unique peptides that had an m/z <1000 containing no missed cleavage sites and no cysteine residues, were sorted for each protein according to their identification in the iTRAQ experiments, and the candidate MRM transitions with 3-5 most intense y (or b) ions for each selected peptide were predicted using Skyline program. The analyses for all the experiments were on a QTRAP 5500 mass spectrometer instrument (AB SCIEX, Foster City, CA, USA) equipped with a Waters nano Acquity Ultra Performance LC system. The in-solution digested and unfractionated peptide mixtures of rubber particle proteins were prepared as described in the Protein Preparation. Approximately 4 µg of the digested sample was injected on a C18 column (3C18-CL-120, 0.075×150 mm column, 3.0 µm, 120A; Eksigent), and the mobile phase consisted of 0.1 % formic acid (FA) in either 2% ACN (A) or 98% ACN (B). Peptides were eluted with a linear gradient of 60 min from 5% B at 0 to 1 min, 5-40% B at 1 to 30 min, 40-80% B at 30 to 45 min, 80% B at 45 to 52 min, 80-5% B at 52 to 52.1 min, and hold in 5% B at 52.1 to 60 min at a flow rate of 0.3 μL/min.

We detected and verified transitions by MRM+EPI mode. The dwell times of two MRM transitions between the precursors and production ions for each peptide were set to 20 ms. The standard protein of β-galactosidase (BG, 24 fmol) was used for instrument correction, and the instrument parameters were optimized to the following settings: ionization spray voltage 2300 V, curtain gas 35 psi, interface heater temperature (IHT) 150°C, declustering potential 100 V, entrance potential 10 V, collision cell exit potential (CXP) 10 V. Quadrupoles (Q1 and Q3) were set to unit resolution. High-purity nitrogen was used as the collision gas in Q2, and the collision energy (CE) for each MRM transition was optimized using Skyline software according to the equation: CE = 3.314 + 0.034 × precursor m/z. ProteinPilot software (Applied Biosystems) was used to search protein database for MS/MS data generated from the MRM-MS assays.

**MRM Quantitative Analysis**

42 serum samples were immunodepleted and digested as previously described. Dried samples were then resuspended in 100μl ACN 0.2% (v/v) formic acid. Each of these samples were prepared in triplicate and processed in a randomized order. These replicate sample preparations were used to determine the CV of the MRM assay for each peptide.

**Western blot**

Myocardial tissues from left ventricle were re-suspended in ice-cold radioimmunoprecipitation (Beyotime, P0013B, China) buffer for lysis. Tissue homogenate were heated with loading buffer and separated using 10% SDS-PAGE and then transferred with Tris–HCl methanol (20 mM Tris, 150 mM glycine and 20% methanol) onto polyvinylidene difluoride membranes (Millipore, USA) in a trans-blot electrophoresis transfer cell (Bio-Rad). After blocking with 5% nonfat milk, membranes were incubated with the primary antibodies rabbit anti-LMNA (1:800) (Abcam incorporation ab26300) or mouse anti-GAPDH (Santa Cruz Biotechnology sc-365062 ) overnight at 4°C. Membranes were further incubated with polyclonal rabit anti-Goat HRP-conjugated secondary antibody (Beijing ComWin Biotechnology CW0103) or polyclonal goat anti-mouse HRP-conjugated secondary antibody (Beijing ComWin Biotechnology CW0102) for 2h at room temperature. Immunopositive bands were visualized using enhanced chemiluminescence reagents (ECL, GE healthcare). Detected bands were quantified with Gel-pro 4.0 software (Media Cybernetics, LP). The relative density of each protein was calculated by dividing the optical density value of each protein by that of the loading control (GAPDH).

**Immunohistochemistry**

Heart sections were de-waxed in xylol, rehydrated in decreasing concentrations of alcohol, and then subjected to microwave antigen retrieval (10 min in 0.1 M citrate acid buffer solution, pH 6). Sections were blocked with 0.3% hydrogen peroxide and PBS containing 10% fetal calf serum (FBS). Sections were then incubated with a rabbit antibody against LMNA (1:100) (Santa Cruz Biotechnology sc-20680) in 10% FBS overnight at 4°C. After washing, sections were incubated with peroxidase-conjugated goat anti-rabbit IgG (Boster Biologics) for 20 min at room temperature and colored using Diaminobenzidine (DAB). Images were taken with a microscope (Nikon, Japan).

**Supplemental Table S1. Differentially expressed proteins identified by iTRAQ analysis**

| Accession no. | | Uniprot_Swissprot Description | NCBI Code | Mascot  score | Coverage (%) | Fold Differences | | | | SignalP  4.1 | | SecretomeP  1.0f |
| --- | --- | --- | --- | --- | --- | --- | --- | --- | --- | --- | --- | --- |
|  |  |  |  |  |  | TOF/C | VSD/C | PTA/C | MIX/C |  |  |  |
| **Proteins lower in CHDs** | | |  |  |  |  |  |  |  |  | |  |
| IPI00021405 | Isoform A of Prelamin-A/C | | LMNA | 138 | 6 | 0.18 | 0.18 | - | 0.21 |  |  |  |
| IPI00418471 | Vimentin | | VIM | 988 | 27.7 | 0.19 | - | - | 0.18 |  |  |  |
| IPI00010779 | Tropomyosin α-4 chain | | TPM4 | 227 | 24.2 | 0.29 | 0.3 | 0.35 | 0.17 |  | |  |
| IPI00029061 | Selenoprotein P | | SEPP1 | 336 | 13.9 | 0.52 | - | 0.64 | - | Yes | |  |
| IPI00298994 | Talin-1 | | TLN1 | 267 | 5.4 | 0.54 | - | - | 0.60 |  | |  |
| IPI00019502 | Myosin-9 | | MYH 9 | 1103 | 10.5 | 0.55 | 0.28 | - | 0.42 |  | |  |
| IPI00925547 | Lactotransferrin | | LTF | 893 | 33.1 | 0.57 | - | 0.53 | - | Yes | |  |
| IPI00418153 | Putative uncharacterized protein | | IGHM | 1859 | 27 | 0.59 | - | - | 0.69 | Yes | |  |
| IPI00784154 | 60 kDa heat shock protein | | HSPD1 | 113 | 9.9 | - | 0.18 | - | - |  | |  |
| IPI00021440 | Actin, cytoplasmic 2 | | ACTG1 | 989 | 40.8 | - | 0.55 | 0.54 | 0.41 |  | |  |
| IPI00478003 | Alpha-2-macroglobulin | | 2M | 3819 | 34.5 | - | 0.57 | - | - | Yes | |  |
| IPI00747752 | V2-7 protein | |  | 199 | 20.5 | - | 0.57 | - | 0.60 | Yes | |  |
| IPI00007244 | Isoform H17 of Myeloperoxidase | | MPO | 255 | 14.2 | - | 0.58 | 0.67 | - | Yes | |  |
| IPI00019359 | Keratin | | KRT9 | 116 | 11.6 | - | 0.60 | - | - |  | |  |
| IPI00010295 | Carboxy peptidase Ncatalytic chain | | CPN1 | 118 | 12.7 | - | - | 0.53 | - | Yes | |  |
| IPI00029168 | Apolipoprotein(a) | | LPA | 134 | 1.3 | - | - | 0.65 | - | Yes | |  |
| IPI00007221 | Plasma serine protease inhibitor | | SERPINA5 | 900 | 30.5 | - | - | - | 0.33 | Yes | |  |
| IPI00302592 | Filamin-A | | FLNA | 408 | 5.5 | 0.59 | 0.32 | - | 0.48 |  | |  |
| IPI00015148 | Ras-related protein Rap-1b | | RAP1B | 88 | 20.7 | - | - | - | 0.51 |  | | Yes |
| IPI00218474 | Isoform 1 of Beta-enolase | | ENO3 | 214 | 13.1 | - | - | - | 0.54 |  | |  |
| IPI00017603 | Coagulation factor VIII | | F8 | 99 | 1.8 | - | - | - | 0.60 | Yes | |  |
| IPI00022389 | Isoform 1 of C-reactive protein | | CRP | 372 | 23.7 | 1.6 | - | - | 0.61 | Yes | |  |
| IPI00023748 | Nascent polypeptide-associated complex subunit alpha | | NACA | 64 | 6.5 | - | - | - | 0.62 |  | |  |
| IPI00011832 | Secreted phosphoprotein 24 | | SPP2 | 173 | 20.9 | - | - | - | 0.64 | Yes | |  |
| **Proteins higher in CHDs** | | |  |  |  |  |  |  |  |  | |  |
| IPI00021855 | | Apolipoprotein C-I | APOC1 | 246 | 30.1 | 9.6 | 8.7 | 8.0 | 10.4 | Yes | |  |
| IPI00975939 | | SAA2-SAA4 protein | SAA2-  SAA4 | 523 | 27.9 | 2.0 | 1.9 | 2.0 | 2.2 | Yes | |  |
| IPI00022295 | | Platelet factor 4 variant | PF4V1 | 314 | 23.1 | 1.9 | 2.7 | 2.5 | 2.2 | Yes | |  |
| IPI00641737 | | Haptoglobin | HP | 909 | 31 | 1.9 | 1.7 | 2.2 | - | Yes | |  |
| IPI00022445 | | Platelet basic protein | PPBP | 379 | 43.8 | 1.6 | - | 2.4 | - | Yes | |  |
| IPI00428732 | | Neurexin-2-β | NRX2B | 73 | 2 | 1.6 | 1.6 | - | 3.2 | Yes | |  |
| IPI00021856 | | Apolipoprotein C-II | APOC2 | 913 | 49.5 | 1.6 | - | - | - | Yes | |  |
| IPI00022389 | | Isoform 1 of C-reactive protein | CRP | 372 | 23.7 | 1.6 | - | - | 0.6 | Yes | |  |
| IPI00025204 | | CD5 antigen-like | CD5L | 1096 | 47.3 | 1.5 | - | - | - | Yes | |  |
| IPI00029260 | | Monocyte differentiation antigen CD14 | CD14 | 237 | 17.6 | - | 2.2 | - | - | Yes | |  |
| IPI00021364 | | Properdin | CFP | 178 | 10.7 | - | 1.7 | - | - | Yes | |  |
| IPI00007199 | | Protein Z-dependent protease inhibitor | SERPINA10 | 488 | 23.4 | - | 1.5 | - | - | Yes | |  |
| IPI00930442 | | Putative uncharacterized protein | IGHG4 | 749 | 19.5 | - | - | 2.6 | - | Yes | |  |
| IPI00022488 | | Hemopexin | HPX | 398 | 21.9 | - | - | 2.6 | - | Yes | |  |
| IPI00303476 | | ATP synthase subunit beta | ATP5B | 139 | 14 | - | - | 2.5 | - |  | |  |
| IPI00021842 | | Apolipoprotein E | APOE | 6249 | 58 | - | - | 2.5 | - | Yes | |  |
| IPI00022463 | | Serotransferrin | TF | 806 | 29.9 | - | - | 2.2 | - | Yes | |  |
| IPI00166729 | | Zinc-alpha-2-glycoprotein | AZGP1 | 67 | 7.4 | - | 1.6 | 2.2 | - | Yes | |  |
| IPI00045109 | | Histone H2A type 1-A | HIST1H2AA | 88 | 24.4 | - | - | 1.8 | - |  | |  |
| IPI00022429 | | Alpha-1-acid glycoprotein 1 | ORM1 | 97 | 19.9 | - | - | 1.8 | - | Yes | |  |
| IPI00064667 | | Beta-Ala-His dipeptidase | CNDP1 | 306 | 28.4 | - | - | 1.6 | - | Yes | |  |
| IPI00298497 | | Fibrinogen beta chain | FGB | 509 | 28.1 | - | - | 1.6 | - | Yes | |  |
| IPI00012269 | | Isoform 1 of Multimerin-1 | MMRN1 | 122 | 2.8 | - | - | - | 1.6 | Yes | |  |

**Supplemental Table S2. Q1/Q3 transitions of the target proteins selected for the MRM experiments**

| **Protein name** | **Peptide sequence** | **MRM Transitions (m/z)** | | **Fragment Ion** | **CE** |
| --- | --- | --- | --- | --- | --- |
|  |  | Q1 | Q3 |  |  |
| β-galactosidase | APLDNDIGVSEATR | 729.37 | 832.45 | y8 | 35.1 |
| β-galactosidase | GDFQFNISR | 542.26 | 636.35 | y5 | 28.4 |
| β-galactosidase | VDEDQPFPAVPK | 671.34 | 755.45 | y7 | 33 |
| Haptoglobin | TEGDGVYTLNNEK | 720.34 | 881.44 | y7 | 34.8 |
| Haptoglobin | TEGDGVYTLNNEK | 720.34 | 718.37 | y6 | 34.8 |
| Haptoglobin | VGYVSGWGR | 490.75 | 661.34 | y6 | 26.5 |
| Haptoglobin | VGYVSGWGR | 490.75 | 562.27 | y5 | 26.5 |
| Isoform A of Prelamin-A/C | IDSLSAQLSQLQK | 715.90 | 915.53 | y8 | 32.6 |
| Isoform A of Prelamin-A/C | IDSLSAQLSQLQK | 715.90 | 844.49 | y7 | 30.6 |
| Isoform A of Prelamin-A/C | IDSLSAQLSQLQK | 715.90 | 603.35 | y5 | 30.6 |
| Isoform A of Prelamin-A/C | IDSLSAQLSQLQK | 477.60 | 716.43 | y6 | 17.6 |
| Isoform A of Prelamin-A/C | IDSLSAQLSQLQK | 477.60 | 603.35 | y5 | 19.6 |
| Isoform A of Prelamin-A/C | IDSLSAQLSQLQK | 477.60 | 516.31 | y4 | 17.6 |
| Isoform A of Prelamin-A/C | VAVEEVDEEGK | 602.29 | 934.40 | y8 | 24.5 |
| Isoform A of Prelamin-A/C | VAVEEVDEEGK | 602.29 | 805.36 | y7 | 26.5 |
| Apolipoprotein C-I | EFGNTLEDK | 526.75 | 719.36 | y6 | 23.8 |
| Apolipoprotein C-I | EFGNTLEDK | 526.75 | 504.27 | y4 | 23.8 |
| Apolipoprotein C-I | EFGNTLEDK | 526.75 | 906.38 | b8 | 23.8 |
| Apolipoprotein C-I | EWFSETFQK | 601.28 | 886.43 | y7 | 30.5 |
| Apolipoprotein C-I | EWFSETFQK | 601.28 | 739.36 | y6 | 30.5 |
| Apolipoprotein C-I | EWFSETFQK | 601.28 | 523.29 | y4 | 30.5 |
| Platelet factor 4 variant | HITSLEVIK | 520.31 | 902.56 | y8 | 27.6 |
| Platelet factor 4 variant | HITSLEVIK | 520.31 | 789.47 | y7 | 27.6 |
| Platelet factor 4 variant | HITSLEVIK | 520.31 | 780.43 | b7 | 27.6 |
| Serum amyloid A-2 protein | EANYIGSDK | 498.74 | 682.34 | y6 | 26.8 |
| Serum amyloid A-2 protein | EANYIGSDK | 498.74 | 519.28 | y5 | 26.8 |
| Serum amyloid A-2 protein | EANYIGSDK | 498.74 | 406.19 | y4 | 26.8 |
| Serum amyloid A-2 protein | SFFSFLGEAFDGAR | 775.87 | 935.46 | y9 | 34.8 |
| Serum amyloid A-2 protein | SFFSFLGEAFDGAR | 775.87 | 822.37 | y8 | 36.8 |
| Serum amyloid A-2 protein | SFFSFLGEAFDGAR | 775.87 | 636.31 | y6 | 36.8 |
| Serum amyloid A-4 protein | EALQGVGDMGR | 566.77 | 819.38 | y8 | 29.3 |
| Serum amyloid A-4 protein | EALQGVGDMGR | 566.77 | 691.32 | y7 | 29.3 |
| Serum amyloid A-4 protein | EALQGVGDMGR | 566.77 | 535.23 | y5 | 29.3 |
| Serum amyloid A-4 protein | AYWDIMISNHQNSNR | 616.95 | 956.43 | y8 | 31.2 |
| Serum amyloid A-4 protein | AYWDIMISNHQNSNR | 616.95 | 869.40 | y7 | 31.2 |
| Serum amyloid A-4 protein | AYWDIMISNHQNSNR | 616.95 | 755.35 | y6 | 31.2 |
| Myosin-9 | ALELDSNLYR | 597.31 | 767.37 | y6 | 30.4 |
| Myosin-9 | ALELDSNLYR | 597.31 | 652.34 | y5 | 30.4 |
| Myosin-9 | ALELDSNLYR | 597.31 | 856.44 | b8 | 30.4 |
| Neurexin-2-β | MPPGGSGPGGCPR | 585.26 | 787.35 | y9 | 27.9 |
| Neurexin-2-β | MPPGGSGPGGCPR | 585.26 | 584.25 | b7 | 31.9 |
| Neurexin-2-β | MPPGGSGPGGCPR | 585.26 | 738.32 | b9 | 31.9 |
| Neurexin-2-β | HGTVPIAINR | 539.31 | 683.42 | y6 | 28.3 |
| Neurexin-2-β | HGTVPIAINR | 539.31 | 586.37 | y5 | 28.3 |
| Neurexin-2-β | HGTVPIAINR | 539.31 | 473.28 | y4 | 28.3 |
| Neurexin-2-β | GGHAGTTYIFGK | 604.81 | 829.45 | y7 | 30.6 |
| Neurexin-2-β | GGHAGTTYIFGK | 604.81 | 627.35 | y5 | 30.6 |
| Neurexin-2-β | GGHAGTTYIFGK | 604.81 | 464.29 | y4 | 30.6 |
| Tropomyosin α-4 chain | SLEAASEK | 417.71 | 634.30 | y6 | 21.9 |
| Tropomyosin α-4 chain | SLEAASEK | 417.71 | 363.19 | y3 | 29.9 |
| Tropomyosin α-4 chain | SLEAASEK | 417.71 | 201.11 | b4 | 17.9 |
| Tropomyosin α-4 chain | LVILEGELER | 585.84 | 958.52 | y8 | 23.9 |
| Tropomyosin α-4 chain | LVILEGELER | 585.84 | 732.35 | y6 | 29.9 |
| Tropomyosin α-4 chain | LVILEGELER | 585.84 | 603.31 | y5 | 23.9 |
| Tropomyosin α-4 chain | IQ[+1_0]ALQQQADEAEDR | 808.38 | 933.39 | y8 | 36 |
| Tropomyosin α-4 chain | IQ[+1_0]ALQQQADEAEDR | 808.38 | 805.33 | y7 | 34 |
| Tropomyosin α-4 chain | IQ[+1_0]ALQQQADEAEDR | 808.38 | 490.23 | y4 | 34 |

CE, Collision Energy; Q1 and Q3, the Q1 and Q3 transitions at m/z, respectively.

**Supplemental Figure**

**
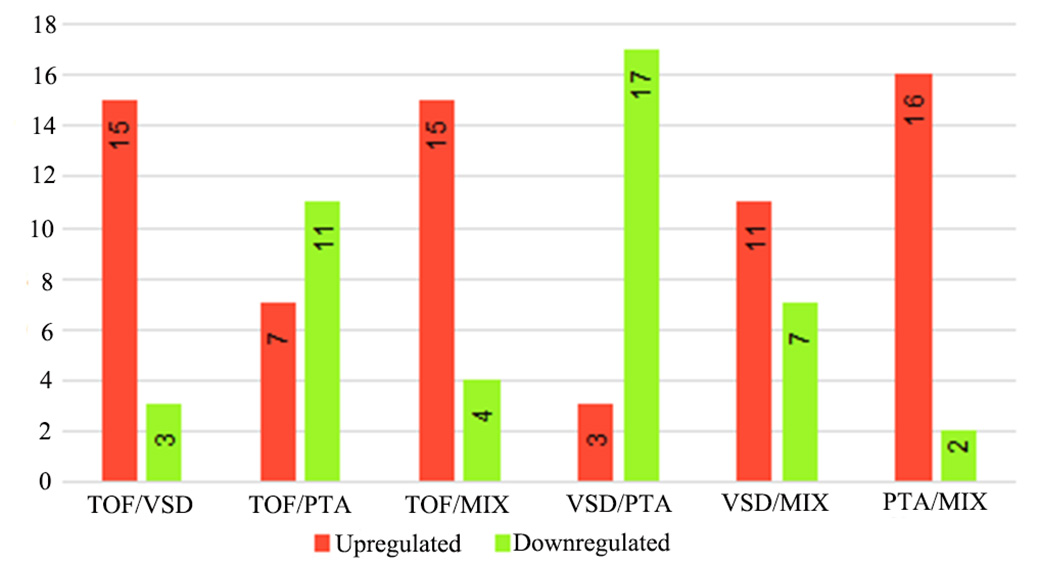
**

**Supplemental Figure S1. Proteins differentially expressed between different subtypes of CHDs.**

**
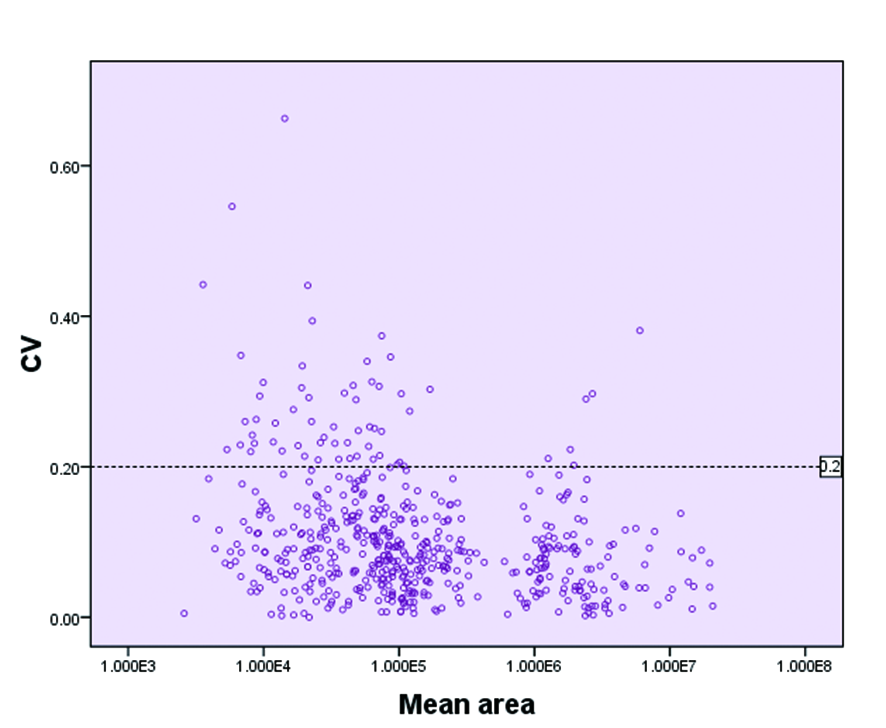
**

**Supplemental Figure S2. CVs of the individual transitions’ peak areas of the 9 proteins over 3 replicates.** Mean area, average of the peak areas over 3 replicates.

**
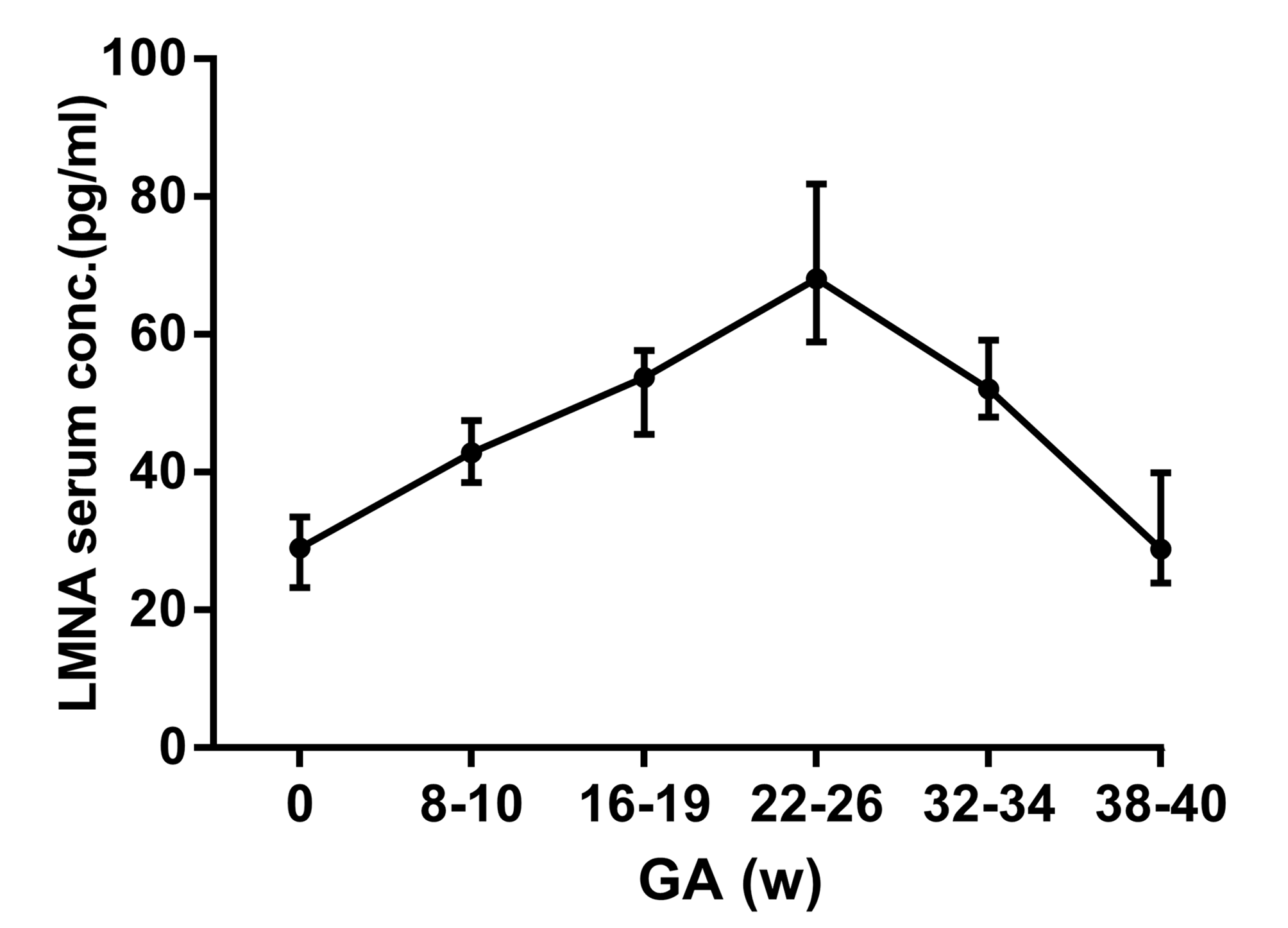
**

**Supplemental Figure S3. Serum LMNA concentrations at different GAs of normal pregnancies.**
